# Consumption of energy drinks by children and young people: a rapid review examining evidence of physical effects and consumer attitudes

**DOI:** 10.1136/bmjopen-2015-010380

**Published:** 2016-10-10

**Authors:** Shelina Visram, Mandy Cheetham, Deborah M Riby, Stephen J Crossley, Amelia A Lake

**Affiliations:** 1 School of Medicine, Pharmacy and Health, Durham University Queen's Campus, Stockton-on-Tees, UK; 2 Fuse (UKCRC Centre for Translational Research in Public Health), Newcastle University, Newcastle-upon-Tyne, UK; 3 School of Health and Social Care, Teesside University, Middlesbrough, UK; 4 Department of Psychology, Durham University, Durham, UK

**Keywords:** child health, systematic review, caffeine, sugar, energy drinks

## Abstract

**Objective:**

To examine patterns of energy drink consumption by children and young people, attitudes towards these drinks, and any associations with health or other outcomes.

**Design:**

Rapid evidence assessment and narrative synthesis.

**Data sources:**

9 electronic bibliographic databases, reference lists of relevant studies and searches of the internet.

**Results:**

A total of 410 studies were located, with 46 meeting the inclusion criteria. The majority employed a cross-sectional design, involved participants aged 11–18 years, and were conducted in North America or Europe. Consumption of energy drinks by children and young people was found to be patterned by gender, with boys consuming more than girls, and also by activity levels, with the highest consumption observed in the most and least sedentary individuals. Several studies identified a strong, positive association between the use of energy drinks and higher odds of health-damaging behaviours, as well as physical health symptoms such as headaches, stomach aches, hyperactivity and insomnia. There was some evidence of a dose–response effect. 2 experimental studies involving small numbers of junior athletes demonstrated a positive impact on limited aspects of sports performance. 3 themes emerged from the qualitative studies: reasons for use; influences on use; and perceived efficacy and impact. Taste and energy-seeking were identified as key drivers, and branding and marketing were highlighted as major influences on young people's consumption choices. Awareness of possible negative effects was low.

**Conclusions:**

There is growing evidence that consumption of energy drinks is associated with a range of adverse outcomes and risk behaviours in terms of children's health and well-being. However, taste, brand loyalty and perceived positive effects combine to ensure their popularity with young consumers. More research is needed to explore the short-term and long-term impacts in all spheres, including health, behaviour and education.

**Trial registration number:**

CRD42014010192.

**Strengths and limitations of this study**

- This is the first independent review of the scientific literature relating solely to the consumption of energy drinks by children and young people.
- Key strengths include the comprehensiveness of the searches, the systematic study selection process and rigorous synthesis methods used.
- The inclusion of qualitative research exploring children and young people's views, alongside quantitative studies on health and other effects, helps to enhance the relevance of the findings for the design and evaluation of future policy and practice interventions.
- The strength of the conclusions is limited by the quality of the individual studies, which varied due to factors such as the sample sizes, cross-sectional designs and reliance on self-report data.
- Few studies examined educational or social outcomes, highlighting a need for further research that examines the short-term and long-term impact of energy drinks in relation to a wider range of outcomes.

## Introduction

Energy drinks are non-alcoholic beverages that typically contain high levels of caffeine (>150 mg/L) and sugar in combination with other ingredients known to have stimulant properties. They are marketed explicitly as a way to relieve fatigue and improve mental alertness, in contrast with sports or isotonic drinks which are intended to help athletes rehydrate after exercise. There are implicit claims that energy drinks promote a more active and healthy lifestyle, in spite of the British Soft Drinks Association (BSDA) pledging that they ‘will not be marketed as sports beverages which deliver a rehydration benefit’.^1^ Between 2006 and 2014, consumption of energy drinks in the UK increased by 155%, from 235 to 600 million L.^2^ This equated to a per capita consumption of 9.4 L and a total value of £1.48 billion. Despite the growing energy drinks market and media reports of serious adverse events associated with their consumption, research into their use and effects has been sparse. In 2011, the European Food Safety Authority (EFSA) commissioned a study to gather consumption data for energy drinks in 16 countries of the European Union.^3^ They found that young people aged 10–18 years had the highest reported consumption prevalence (68%), compared with adults over 18 years (30%) and children under 10 years (18%). On average, young people in the UK were found to consume more energy drinks than their counterparts in other EU countries (3.1 L/month in comparison with 2.1 L).

The scientific literature focuses largely on the effects of energy drink consumption in adults, who may experience temporary benefits such as increased cognitive performance, enhanced mood, more physical energy and promotion of wakefulness.^4–7^ However, evidence is emerging on the harmful physiological and psychological effects of these drinks, and it is possible that prolonged use may affect physical and mental well-being.^8^ With children and young people, anecdotal evidence suggests that those who regularly consume energy drinks can become dependent on them and even moderate consumption may be detrimental.^9–11^ Based on the known effects of caffeine, consumption of energy drinks may lead to: caffeine intoxication and withdrawal; sleep disruption and insomnia; and disruptive, hyperactive and risky behaviour.^12–14^ There are also likely to be longer term health implications associated with excessive sugar intake, such as dental erosion, obesity and type 2 diabetes.^15–18^

Based on the importance and impact of energy drink consumption outlined above, the objectives of this study were to review the existing literature in order to: (1) examine evidence of associations (if any) between children and young people's consumption of energy drinks and their health and well-being, social, behavioural or educational outcomes; (2) determine whether the magnitude and direction of these associations vary according to the quantity or frequency of energy drinks consumed and (3) explore children and young people's attitudes towards energy drinks and, in particular, what factors motivate them to consume or to abstain from consuming these drinks.

## Methods

We undertook a time-limited review of the literature following the guidance on rapid evidence assessments (REAs).^19^ REAs aim to be rigorous and explicit in method, but make concessions to breadth or depth by limiting particular aspects of the review process; for example, less exhaustive use of ‘grey’ sources and a preference for electronically available texts. Rapid or pragmatic reviews have been shown to produce similar results, more quickly and at a lower cost when compared with systematic reviews, suggesting that the approach employed here was useful and valid.^20^ ^21^

### Search methods

Searches of the following major bibliographic databases were undertaken: ASSIA, CINAHL, Cochrane Library, DARE, EMBASE, ERIC, MEDLINE, PsycINFO and Web of Science. We also conducted searches of OpenGrey and the internet using Google to locate grey literature. Specific search strategies were employed for each database. See box 1 for lists of the key terms used. The results of each search were exported to an independent database using EndNote 7 software. These databases were subsequently merged into a single unique database and duplicates were removed automatically.

Box 1 **Search terms**

List 1: topic

‘Energy drink’ OR ‘stimulant drink’ OR ‘energy shot’ OR ‘energy strip’ OR ‘energy mint’

List 2: population

Child* OR adolesc* OR teen* OR (young AND (person OR people)) OR youth

List 3: outcomes

‘Health effect’ OR ‘adverse effect’ OR ‘positive effect’ OR wellbeing OR ‘physical energy’ OR wakeful* OR alert* OR ‘mental boost’ OR performance OR sleep OR insomnia OR mood OR depress* OR anxi* OR (caffeine AND (intoxication OR withdrawal)) OR dehydrat* OR headache OR nausea OR pain OR stress OR weight OR BMI OR ‘body mass index’ OR ‘metabolic rate’ OR ‘blood sugar’ OR ‘blood pressure’ OR ‘heart rate’ OR cardiovascular OR (dental AND (health OR erosion OR caries)) OR ((disruptive OR risky OR hazardous OR anti-social OR criminal) AND behavio*) OR ADHD OR ADD OR ‘attention hyperactivity deficit disorder’ OR drug OR alcohol OR smok* OR ‘screen time’ OR ‘physical activity’ OR exercise OR sport OR sedentary OR sex OR ‘self-harm’ OR violence OR injury OR sociability OR ‘peer effects’ OR learning OR memory OR attention OR attainment OR achievement OR ((absence OR exclusion) NEAR school)

We adopted an inclusive approach to locating original and review articles published between January 2000 and April 2016 that examined the consumption of energy drinks by children and young people aged 0–18 years (or up to 20 years if still in secondary education). This period was chosen to reflect the time when major brands became widely available; for example, Red Bull was introduced in the USA in 1997 and Monster was created in 2002. The inclusion of studies was not restricted according to outcome type or study setting, although we excluded studies that only involved college or university students. Animal studies, articles not published in English and studies focusing on individual ingredients (eg, caffeine or taurine) rather than energy drinks as a whole were excluded. We also excluded opinion pieces, editorials and descriptive papers without empirical findings.

### Study selection

**Figure 1:**
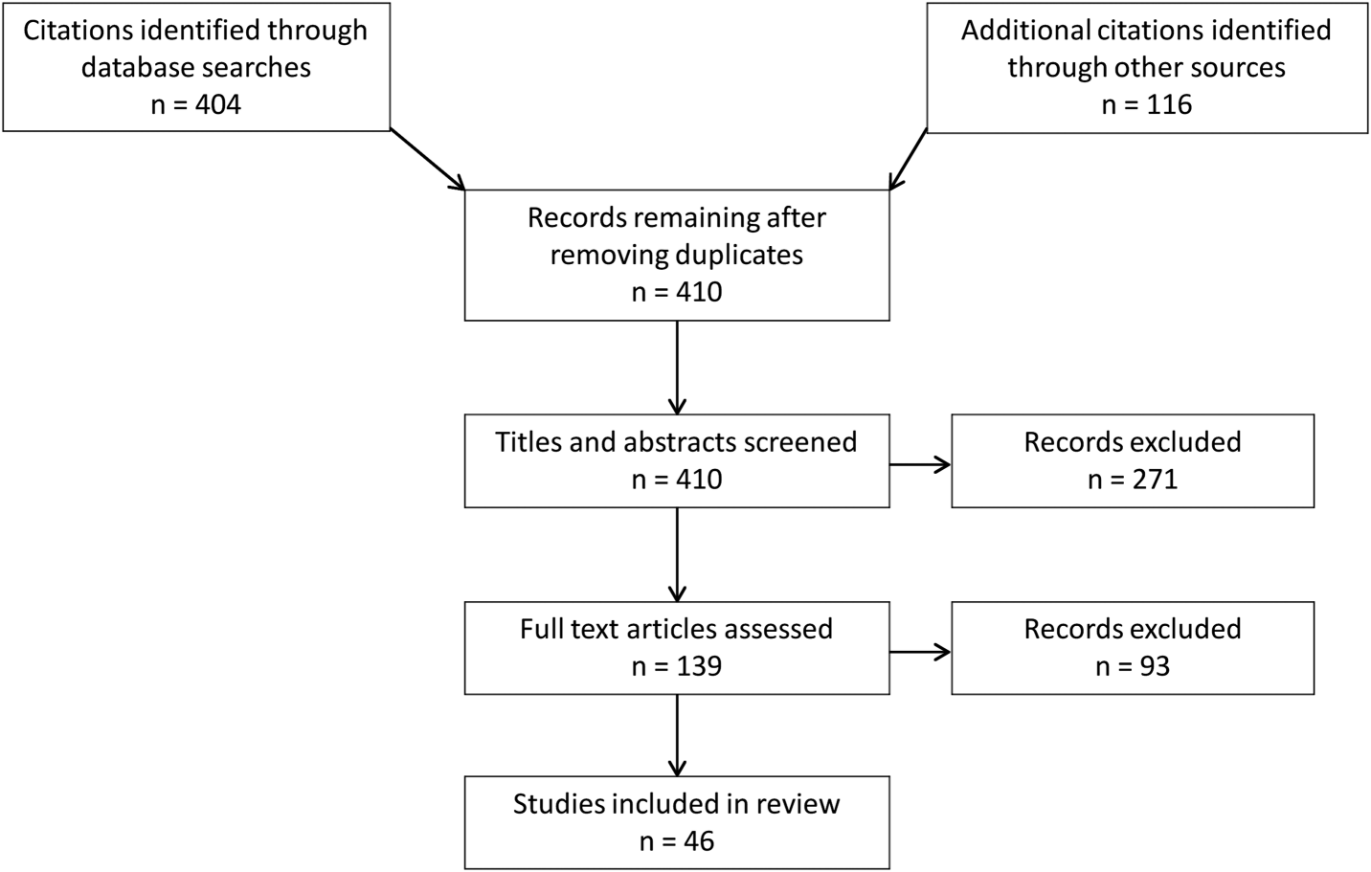
Study selection flow chart.

Titles of studies identified from the searches were scanned by two researchers to make an initial assessment of relevance. In cases where there was any doubt, abstracts were retrieved in order to make a further judgement. Where possible, we obtained the full text of all references included after the title/abstract screening stage and PDF files were added to the bibliographic database. Articles deemed potentially relevant were reviewed independently by two researchers based on the inclusion criteria. Any disagreements (26 in total) were resolved by discussion between the researchers, with referral to a third member of the team where necessary. Information regarding the eligibility of a reference—relevant or not relevant—was recorded in the database to monitor the screening process. The process is summarised in the study selection flow chart (figure 1).

### Quality assessment

Formal appraisal of eligible studies was undertaken using the Quality Assessment Tool for Quantitative Studies developed by the Effective Public Health Practice Project (EPHPP) and the Critical Appraisal Skills Programme checklist for qualitative studies.^22^ ^23^ Both checklists have been widely used in previous systematic reviews and allow for rapid evaluation of study quality. Each paper was independently appraised by two researchers and disagreements (four in total) were resolved through discussion to reach an overall judgement concerning the quality of the available evidence (strong, moderate or weak). Studies rated as weak were excluded from the review. The six previous reviews located through our searches were either judged to be of insufficient quality to provide reliable evidence or they included studies involving adults or non-human animals, making it difficult to extract results relating specifically to children and young people.^12^ ^13^ ^24–27^ However, they contributed to the identification of relevant primary studies.

### Data extraction

Eligible studies were divided between members of the research team. Data were then extracted onto a template by the lead researcher for each study. Variables to be extracted included: bibliographic information; country of origin; study setting; methods; participant characteristics; outcomes; time frame; and results of the critical appraisal. This information was stored in a Microsoft Access database. As the object was to explore all possible health, behavioural, educational and social impacts of energy drink consumption, data were extracted on any outcomes and measures used in the studies.

### Data synthesis

A quantitative synthesis proved to be inappropriate due to the heterogeneity of study designs, contexts and outcomes. Data from all studies that met the inclusion and quality criteria have therefore been descriptively summarised and narratively synthesised. Narrative synthesis relies primarily on the use of text rather than statistics to ‘tell the story’ of the findings from the included studies.^28^ This method is often used to increase the chances of the findings of a review being used in policy and practice. In the present review, the main narratives concerned the reported effects of energy drink consumption and the experiences and attitudes of young consumers, clearly related to the stated objectives.

## Results

### Study characteristics and quality

Forty-two quantitative studies and four qualitative or mixed method studies met our inclusion criteria. The quantitative studies included 31 cross-sectional surveys, four longitudinal studies, four papers reporting retrospective analyses of national or regional poison centre data and three experimental studies (tables 1–4). Most (n=38) explored the use of energy drinks by young people aged between 11 and 18 years. Studies were largely conducted in North America (n=22) or Europe (n=12), yet all of the qualitative studies were from Australia or New Zealand (table 5). Other study contexts included the Middle East (n=5) and South America (n=1). Equal numbers of studies were rated as being of strong or moderate quality (n=23 each).

**Table 1:** Experimental studies

Citation	Type	Quality assessment	Country	Participants	Study design	Intervention and dose	Outcome measures	Main findings
Abian-Vicen *et al* (2014)^67^	Full paper	Strong	Spain	Boys (n=16) Mean 14.9±0.8 years	Double-blind, placebo-controlled experimental design with repeated measures	A commercially available ED (dose: 3 mg caffeine/kg body weight)	Jump performance, power, endurance and shot precision in highly skilled basketball players	Significant increases in: jump height, mean leg muscle power output, perceived muscle power, endurance and vigour during the hours following the test Decreased rate of perceived exhaustion No difference in: precision of basketball shots, total number of free throws per second or distances covered
Gallo-Salazar *et al* (2015)^66^	Full paper	Strong	Spain	Boys and girls (n=14) Mean 16 ±1 years	Double-blind, placebo-controlled experimental design with repeated measures	A commercially available ED (dose: 3 mg caffeine/kg body weight)	Physical performance in elite junior tennis players	Significant increases in: handgrip force, running pace at high intensity, and number of sprints during a simulated match No difference in: peak running speed; ball velocity during the serving test Sweat rate during the simulated match was slightly increased, producing significantly higher dehydration
Temple *et al* (2010)^37^	Full paper	Strong	USA	Boys and girls (n=52) 12–17 years	Double blind, placebo-controlled experimental design	Drinks containing 0 mg, 50 mg, 100 mg or 200 mg of caffeine	Cardiovascular and subjective responses to caffeine and snack food ingestion	Dose-dependent increases in diastolic blood pressure (DBP) and decreases in HR In boys, high-caffeine consumers showed greater increases in DBP over time than did low-consuming boys High-caffeine consumers had more energy, protein and fat in their typical diet

**Table 2:** Retrospective studies

Citation	Type	Quality assessment	Country	Participants	Study design	Outcome measures	Main findings
Gunja and Brown (2012)^65^	Full paper	Moderate	Australia	Boys and girls (n=62) All ages	Retrospective review of NSW Poisons Information Centre data (January 2004–December 2010)	Calls relating to caffeinated ED ingestion	62 children were reported to have accidentally ingested EDs (mean age 36 months, range 7–120 months) 14 had symptoms probably related to EDs (most commonly hyperactivity) and 9 required hospital assessment
Hernandez *et al* (2009)	Conference abstract	Moderate	USA	Boys and girls (n=428) All ages	Retrospective statistical analysis of Texas regional poison centres data	Calls relating to ED ingestion, negative health consequences	The largest affected group was teenagers (n=114, compared with 84 cases <5 years) Significant increases were noted between 2000 and 2001 (+100%); 2003–2044 (+87.5%) and 2005–2006 (+85%) Major symptoms were: rapid heart rate, nervousness/agitation, nausea, vomiting, upset stomach, dizziness, tremors, chest discomfort and headache
Seifert *et al* (2011)^13^	Full paper	Moderate	USA	Boys and girls (n=1568) All ages	Retrospective analysis of US National Poison Data System	Exposure to EDs, adverse health events (toxicity)	Single product, caffeine-containing exposures disproportionately involved those aged <20 years (particularly males) compared with other substance exposures Age groups in this category were: 47% children <6 years, 13% 6–12 years, 19% 13–19 years, 12% 20–25 years, 9% >25 years 13–19-year-olds had the highest proportion of moderate or major effects (19%); the latter included cardiac disturbances, hypertension and hyperthermia
Seifert *et al* (2013)^64^	Full paper	Moderate	USA	Boys and girls (n=4854) All ages	Retrospective analysis of US National Poison Data System	ED use and ED-related toxicities	Of the 4854 calls relating to ED exposure, 3192 cases were categorised as ‘unknown’, 1480 were non-alcoholic and 182 alcoholic Children under 6 had the highest proportion of unintentional exposures to non-alcoholic ED; minor or moderate adverse effects were reported Adolescents (13–19) had the highest proportions of intentional exposures and the largest proportion of cases of minor to moderate effects, with one major effect For alcoholic ED, 54.3% ingestions were 13–19 years, 4.1% 6–12 years and 9.8% <6 years; a greater proportion of alcoholic ED cases were advised to seek treatment

**Table 3:** Cross-sectional studies

Citation	Type	Quality assessment	Country	Participants	Study design	Outcome measures	Main findings
Al-Hazzaa *et al* (2014)^62^	Full paper	Moderate	Saudi Arabia	Boys and girls (n=2908) 15–19 years	Multicentre cross-sectional study	Weight, height, BMI, total daily screen time (ST), physical activity (PA) and dietary habits (DH) using self-report questionnaires	Significant associations of higher consumption of EDs with higher PA levels and higher ST PA did not correlate with consumption of sugar-sweetened drinks overall but did associate significantly with intake of EDs Insignificant associations between PA and intake of EDs in females
Arria *et al* (2014)^51^	Full paper	Moderate	USA	Boys and girls (n=12 267 at T1, n=12 381 at T2) 13–14, 15–16 and 17–18 years	Analysis of data from the 2010 and 2011 Monitoring the Future (school-based) Survey	Use of EDs and energy shots, sociodemographic variables	Younger students, males and Hispanic individuals were more likely to drink EDs. Consumption of energy shots was less prevalent than for EDs and ethnic variations were less apparent, although the gender differences were similar. Between 8% and 12% of students consumed EDs and energy shots. Results were largely consistent between 2010 and 2011
Azagba *et al* (2013)^48^	Full paper	Strong	Canada	Boys and girls (n=36 155) 12–18 years	Cross-sectional, classroom-based, biennial Youth Smoking Survey	Consumption, mixed or premixed with alcohol during the past 12 months	About 20% reported using alcohol mixed with EDs in the last year; prevalence of use was highest among Aboriginal (33.8%) and black (25%) students Students who were older; currently smoked; were involved in heavy drinking in the past year; used marijuana in the past year; were absent from school; participated in school team sports; and had $40 or more weekly spending money were more likely to consume alcohol mixed with EDs in the previous year Students who felt more connected to school and who had an academic average of 70% or higher were less likely to consume EDs
Azagba and Sharaf (2014)^55^	Full paper	Strong	Canada	Boys and girls (n=15 875) 14–18 years	Cross-sectional, classroom-based, biennial Youth Smoking Survey	Susceptibility to smoking, consumption of alcohol mixed with ED (AmED)	About 13% of students used AmED A statistically significant positive association was identified between consuming AmED and susceptibility to smoking among adolescent students Never-smoking students who reported consuming AmED had higher odds of susceptibility to smoking
Azagba *et al* (2014)^29^	Full paper	Strong	Canada	Boys and girls (n=9226) 12–13 years, 14–16 years, 17–18 years	Cross-sectional, high school-based Student Drug Use Survey	ED usage, substance use, sensation-seeking	62% reported consuming EDs at least once in the previous year, with about 20% reporting use once or more per month Sensation-seeking, depression, and substance use were all higher among ED users relative to non-users, and in higher frequency users relative to lower frequency users Males were much more likely to report ED consumption than female students Rates of ED use were higher among younger students and the prevalence of consumption decreased with age
Cotter *et al* (2013)^56^	Full paper	Moderate	USA	Boys and girls (n=43) 13–17 years	Cross-sectional computerised questionnaire with subcritically ill or injured adolescents in paediatric emergency department	Prevalence, quantity and coingestation of alcohol, caffeinated beverages, pills, illicit drugs and tobacco use over previous 30 days and lifetime usage Reasons for ED use	ED drink use among adolescents far exceeded that of alcohol, ‘street’ or illicit drug and tobacco usage. Those who reported ED use had higher prevalence of street drug use than non-ED users On a typical day, adolescents drank 1.5 EDs (range, 0–7 drinks) The most highly cited reasons for ED use were: 29.4% ‘to play sport better’, 23.5% ‘to keep awake for school’, 23.5% ‘because your friends or others were using them’, 23.5% ‘to party’, 11.8% ‘to lose weight’ (less cited reasons: 5.9% ‘to not feel hungry’, ‘to keep awake for work’, ‘to do better at work’, ‘to keep awake while driving’)
Emond *et al* (2014)^38^	Full paper	Moderate	USA	Boys and girls (n=3342) 15–23 years	Cross-sectional national survey conducted by phone	ED and alcohol use (AUDIT scale), demographics, sensation-seeking	16.2% had consumed EDs at least once in the past 7 days. Older participants, males and those with a higher propensity for sensation-seeking were more likely to have recently consumed EDs Alcohol use was more common among those who reported ED use in the past 7 days (80.1%) compared with those who did not (59.9%). After adjusting for sex, ethnicity, number of friends who drink, parental drinking frequency and sensation-seeking, this association remained for 15–17 years only
Evren and Evren (2015)^39^	Full paper	Strong	Turkey	Boys and girls (n=4957) 15–16 years (mean 15.6 years)	Cross-sectional, classroom-based online survey	Demographics, school life and performance, psychological trauma, psychological and behavioural problems, substance use	ED consumption was associated with being male, lifetime substance use, sensation-seeking, psychological problems and self-destructive behaviour. In most cases, there was evidence of a dose–response effect There was no significant association between ED use and age
Faris *et al* (2015)^49^	Full paper	Moderate	Saudi Arabia	Boys only (n=1006) 12–18 years	Cross-sectional, school-based, multiple choice, validated questionnaire	ED use, nutritional and lifestyle factors	60.2% consumed EDs. Frequency and quantity of consumption were both significantly higher in secondary school students than middle school students. Higher disposable income and poor lifestyle behaviours (irregular sleep, smoking, drinking alcohol, low physical activity and unsafe behaviours such as not wearing a seatbelt while driving) were also associated with ED consumption Poor knowledge concerning ED composition was reported. Physiological effects reported by consumers were reduced sleeping hours (23.6%), changes in cardiac activity (18.9%) and becoming energised/activated (16.6%)
Gallimberti *et al* (2013)^47^	Full paper	Moderate	Italy	Boys and girls (n=913) 11–13 years	Cross-sectional school-based survey	Consumption of EDs, other substance abuse	Use of EDs increased significantly with age, from 17.8% among sixth graders to 56.2% among eighth graders Among the male student population, 16.5% of those in the eighth grade and 6.21% of those in the sixth grade drank them at least once a week Independent variables conferring a higher likelihood of being at least once-a-week ED consumers were smoking and alcohol consumption. Awareness of the damage caused by EDs emerged as a protective factor that reduced the likelihood of young students consuming such drinks
Gallimberti *et al* (2015)^40^	Full paper	Moderate	Italy	Boys and girls (n=1496) 10–16 years	Cross-sectional, school-based survey, from 76 classes	Alcohol and substance use and abuse	Smoking, alcohol and ED use increased with age. ED use was more common in males, with the exception of those in the fifth grade. Lifetime ED consumption in the eighth grade was 64.0% and 36.4% for males and females, respectively
Gambon *et al* (2011)^30^	Full paper	Moderate	The Netherlands	Boys and girls (n=502) 12–19 years	Cross-sectional school-based survey, single centre	Data on consumption of EDs, soft drinks, sports drinks and alcopops	39.4% used EDs (in comparison with 85.2% soft drinks, 44.7% sports drinks, 12.8% alcopops) Boys consumed soft drinks, EDs and sports drinks more frequently than girls, and on average also consumed higher amounts of these drinks Significant positive associations were observed between the consumption of soft drinks, EDs and/or sports drinks. Alcopop consumption was only associated with EDs The mean consumption of soft drinks, EDs and sports drinks was highest at 14–15 years, after which it declined
Hamilton *et al* (2013)^57^	Full paper	Strong	Canada	Boys and girls (n=4472) 12–19 years	Retrospective review of Ontario Student Drug Use and Health Survey	ED intake	49.6% of adolescents had consumed EDs in the previous year Energy drink consumption in the previous year was highly associated with tobacco, cannabis and non-medicinal use of prescription drugs use in the previous year, and binge drinking in the previous month. Consumption was also highly associated with sensation-seeking and self-reports of medical treatment for an injury (reported by 16% and 42% of adolescents)
Huhtinen *et al* (2013)^31^	Conference abstract	Moderate	Finland	Boys and girls (n=10 406) 12–18 years	Adolescent Health and Lifestyle Survey, postal and online survey	Association between EDs and four caffeine-induced health symptoms (headache, sleeping problems, irritation, tiredness/fatigue)	44% of adolescents used EDs at least sometimes (2% of girls and 4% of boys used them daily, 0.5% several times a day) Daily use of EDs was strongly associated with the four health symptoms. In adjusted models, health symptoms among those who used EDs several times a day were multifold compared with the non-users: headache (OR=4.5), sleeping problems (3.5), irritation (2.4) and tiredness/fatigue (3.4).
Ilie *et al* (2015)^41^	Full paper	Moderate	Canada	Boys and girls (n=10 272) 11–20 years	Population-based, cross-sectional school survey (Ontario Student Drug Use and Health Survey)	Traumatic brain injuries (TBIs), ED and alcohol use	Gender, alcohol use, ED consumption, AmED and academic performance were identified as significant predictors of TMI. The odds of sustaining a lifetime or recent TBI increased with consumption of alcohol and EDs
Koivusilta *et al* (2016)^63^	Full paper	Moderate	Finland	Boys and girls (n=9446) 13 years	Cross-sectional, classroom based survey	Frequency of ED consumption, health symptoms, time of going to bed on school days	The percentage of adolescents suffering from health symptoms (headache, irritation or outburst of anger, trouble falling asleep or waking at night, tiredness/fatigue) or going to bed late increased with increasing frequency of ED consumption. The relationship between EDs and health symptoms was partly mediated through going to bed late. Results were similar for both genders
Kristjansson *et al* (2014)^42^	Full paper	Strong	Iceland	Boys and girls (n=11 267) 10–12 years	Population-based primary school survey	Prevalence of caffeinated sugar-sweetened beverages (CSSBs) and the relationship with common physical symptoms	Just over 7% of boys and almost 3% of girls reported consuming EDs on a daily basis Use of CSSBs was more common among boys and physical symptoms were more common among girls. About one in five girls reported having headaches, stomach aches and/or sleeping problems sometimes or often during previous 7 days. The prevalence of physical symptoms generally increased for both genders with greater ED use
Kumar *et al* (2014)^32^	Full paper	Strong	USA	Boys and girls (n=840) 12–17 years	Online survey	ED consumption	9% reported consuming ED ≥1 time/week Significant differences were found by age and gender (increasing prevalence among older teens and in males) but not for the other characteristics examined Only 11.5% were ever asked by their doctor/nurse about how often they drank EDs, and 11.1% were ever recommended by their doctor/nurse to not drink EDs The proportion of youth who consumed energy drinks ≥1 time/week was higher among youth who were asked by their doctor/nurse about how often they drank energy drinks than by youth who were not
Larson *et al* (2014)^33^	Full paper	Strong	USA	Boys and girls (n=2793) Mean 14.4 years (SD 2.0 years)	Cross-sectional school-based survey (questionnaire plus anthropometric measures)	Sport and ED intake, PA and sport participation, media use, sleep, cigarette smoking, breakfast frequency and other beverage intake, weight status, demographics	Overall, EDs were consumed at least 1/wk by 14.7% of the sample (significantly higher among boys than girls). Differences in ED consumption by ethnicity were statistically significant only among girls Regular ED consumption was associated with measures of media use, other beverage intake and cigarette use, but was unrelated to measures of PA. For both genders, regular consumption was positively associated with ever having smoked cigarettes and weekly video game use There was a significant association between regular ED consumption and higher daily intake of sugar-sweetened soft drinks and fruit drinks. For girls only, there was also a significant association with lower frequency of breakfast
Locatelli *et al* (2012)^34^	Full paper	Strong	Brazil	Boys and girls (n=2705) 15–17 years	Self-administered questionnaire in private high schools	Alcohol use by socioeconomic level and gender	31.6% reported having used alcohol and ED together at least once in life. Boys reported a higher prevalence of frequent alcohol use, binge drinking and the combination of alcohol with ED Mixing alcohol and ED was most common in students from class A1 (45.5%) and decreased gradually with socioeconomic class to 17.7% in classes D/E (high to low)
Lubman *et al* (2014)^59^	Full paper	Moderate	Australia	Boys and girls (n=558) 17–18 years	Breathalyser tests and brief ‘on street’ surveys	Alcohol, ED and illicit drug use, experience of aggressive incidents, alcohol-related injury and unprotected sex	Those who coconsumed alcohol and energy drinks (one in six participants) recorded a significantly higher blood alcohol content (11.34 vs 8.30), reported feeling more intoxicated and were rated as more intoxicated by interviewers than alcohol-only users
Bryant Ludden and Wolfson (2010)^36^	Full paper	Moderate	USA	Boys and girls (n=197) 14–18 years	Self-report measures completed during school hours	Patterns of caffeine use, linking to reasons for use, expectancies and sleep patterns	Among those who used caffeine the previous day, 6.1% reported ED use (compared with 60.5% soda, 19.3% coffee, 4.4% tea and 8.8% other) Males drank soda and ED more frequently, although females were more likely to report withdrawal/dependence caffeine expectancies and appetite suppression expectancies
Magnezi *et al* (2015)^50^	Full paper	Moderate	Israel	Boys and girls (n=802) 14–18 years	School-based survey	ED and alcohol mixed with ED (AmED) consumption	84.2% had ever drunk EDs. Consumption was more common among older students, immigrants, those from single parent families, and boys, who were more likely than girls to drink them daily. Those who began drinking at an earlier age were more likely to consume AmED 50.2% drank EDs because of the taste, 12.7% to feel energised, 19.3% in order to mix with alcohol, 11% to stay awake and 5.3% reported drinking out of curiosity. More than half knew that EDs mask the effect of alcohol and most knew that those who drink AmED drink more alcohol than those who do not mix it with ED
Musaiger and Zagzoog (2014)^35^	Full paper	Moderate	Saudi Arabia	Boys and girls (n=1061) 12–19 years	School-based short questionnaire extracted from a validated questionnaire, after modifications to include ED	Knowledge, attitudes and intake of energy drinks among adolescents	31.9% of males and 24.7% of females drank EDs 1–2 days/week, with a significant difference between the genders Advertisements were the main single source of information on ED. The main reasons for consumption were for their taste and flavour (58.4%), in order to ‘try them’ (51.8%) and to ‘get energy’ (43%) About half did not know the ingredients of ED and a similar proportion knew they contained caffeine. Two-thirds viewed EDs as soft drinks
Nowak and Jasionowski (2015)^43^	Full paper	Moderate	Poland	Boys and girls (n=2629) 12–20 years (mean 15.8 years)	Classroom-based survey	Demographics, self-reported weight, height, participation in sports, use of EDs, knowledge of contents and their effects	67% participants drank EDs. Use was significantly more common among boys, those who played sport and younger students, although older participants were more likely to mix EDs with alcohol Consumers reported using EDs for no particular reason (21%), when feeling tired (18%), before physical effort (13% and 10%) when thirsty (12%) and while at parties (10%). Around one-third believed EDs were bad for their health and 7% admitted feeling some discomfort after drinking an ED. The most common health problems were: stomach ache (46%), anxiety and heart palpitations (15%), and nausea and vomiting (15%). Nearly, 27% felt overexcited after drinking an ED
Park *et al* (2012)^52^	Full paper	Strong	USA	Boys and girls (n=11 209) 14–18 years	School-based survey	Demographic characteristics, weight status, availability of school vending machines, and behavioural factors with sugar-sweetened beverage (SSB) intake	Mean total ED intake was 0.2 times per day and only ∼5% of students reported drinking a can, bottle or glass ≥1 time per day. Being male, non-Hispanic black, or Hispanic (vs non-Hispanic white), eating at fast-food restaurants ≥3 time per week and watching television >2 hours/day were significantly associated with greater odds of drinking EDs ≥1 time/d, whereas having beverage vending machines in the school was significantly associated with reduced odds of drinking EDs ≥1 time/day
Reid *et al* (2015)^44^	Full paper	Strong	Canada	Boys and girls (n=23 610) 14–18 years	Findings from year 1 of a retrospective cohort study (COMPASS) involving a school-based survey	ED and alcohol use, sociodemographic variables	The odds of using EDs were significantly greater in the following groups: males, off-reserve Aboriginal students, and students reporting some spending money. Among males, ED use increased with age, while the opposite was true for females. Students with a healthy BMI were less likely to report consuming EDs than those who were underweight or obese Intensity of alcohol use was strongly associated with ED use. Binge drinking was the strongest predictor of using alcohol mixed with EDs
Schwartz *et al* (2015)^45^	Full paper	Strong	USA	Boys and girls (n=1649) 10–11, 12–13 and 13–14 years (mean 12.4 years)	30 min online, classroom based health survey	Hyperactivity/Inattention subscale of the Strengths and Difficulties questionnaire, number and types of sweetened beverages	Boys reported drinking significantly more EDs. Black and Hispanic students were more likely to report consumption of EDs. Students who reported consuming EDs were 66% more likely to score in the at-risk category on the hyperactivity/inattention subscale compared with students who did not, regardless of overall sweetened beverage intake. For each additional sweetened beverage, the odds of being at risk for hyperactivity/inattention increased by 14%. After adding beverage types to the model, only EDs had an independent association with risk of hyperactivity/inattention, even after adjusting for number of drinks consumed and other potential confounders.
Terry-McElrath *et al* (2014)^14^	Full paper	Strong	USA	Boys and girls (n=21 995) 13–18 years	Classroom based self-completion questionnaire	ED consumption and substance use	ED/shot use was higher among boys, younger students and those residing outside of metropolitan areas. There were negative relationships with two parents in the home and higher average parental education. Neither race/ethnicity nor region was associated with ED/shot use and consumption did not significantly change between 2010 and 2011 ED/shot use frequency was significantly and positively correlated with past 30-day use frequency of all substance use measures (alcohol, cigarettes, marijuana and amphetamines) for all grades
Van Batenburg-Eddes *et al* (2014)^61^	Full paper	Strong	The Netherlands	Boys and girls (n=509) 11–16 years (mean 13.1 years, SD 0.85)	Cross-sectional school-based survey, part of a larger longitudinal project	Executive functions, plus caffeine and ED intake	6% reported consuming on average at least one ED a day. Problems with falling asleep and waking up were reported most often (23%). Consuming on average one ED or more a day was associated with problems with self-reported behaviour regulation. Participants who drank at least two consumptions of caffeine or ED also had more problems with metacognitive skills
Vilija and Romualdas (2014)^54^	Full paper	Strong	Lithuania	Boys and girls (n=1747) 12–13 years	Cross-sectional school-based study using self- administered questionnaire	Post-traumatic stress (PTS) symptoms, lifetime traumatic experiences, food frequency scale, sense of coherence scale	21.0% consumed EDs on a daily basis. All lifetime traumatic events were associated with unhealthy foods (including EDs) and sense of coherence weakened the strength of the associations

**Table 4:** Longitudinal studies

Citation	Type	Quality assessment	Country	Participants	Study design	Outcome measures	Main findings
Choi *et al* (2016)^58^	Short paper	Moderate	USA	Boys and girls (n=894 at T1, n=780 at T2) 16–18 years (mean 17.0 years, SD 0.77)	Longitudinal mixed mode survey (web, telephone and paper) at baseline and 12 months	Alcohol and ED use, binge drinking	ED use was positively associated with alcohol use. After controlling for alcohol use at baseline, the effect size of ED use in the past month decreased or became non-significant. ED use at baseline predicted the number of drinking days, but not past month binge drinking or average drinks per drinking day, at follow-up
Martz *et al* (2015)^60^	Full paper	Moderate	USA	Boys and girls (n=6498) 17–18 years	Monitoring the Futures surveys completed by 12th grade students in 2012 and 2013	Use of alcohol mixed with EDs (AmED), academic and social factors, other substance use, unsafe driving	Males had significantly greater odds of any AmED use than females, and White or Hispanic students had significantly greater odds than Black students. AmED use was significantly associated with greater odds of driving violations and accidents after alcohol use, controlling for all other variables
Miyake and Marmorstein (2015)^53^	Full paper	Moderate	USA	Boys and girls (n=144 at T1, n=127 at T2) 11–13 years	Classroom-based survey at baseline and 16 months	Use of caffeinated drinks and alcohol, sensation-seeking, parental monitoring	Frequency of ED consumption at baseline predicted increases in frequency of alcohol consumption 16 months later. Lower levels of parental monitoring were associated with higher levels of ED consumption and later frequency of alcohol use There were significant associations between baseline levels of sensation-seeking and frequency of ED consumption, but not later alcohol use
Richards and Smith (2016)^46^	Full paper	Strong	UK	Boys and girls (n=2610 at T1, n=2307 at T2) 11–16 years	Longitudinal study of diet in secondary school children, using the Diet and Behaviour Scale, at baseline and 6 months	Dietary intake of common foods and drinks, exercise frequency, and self-assessed mental health (at T2 only)	Drinking EDs once a week or more was significantly associated with being male, older, having special educational needs and being eligible for free school meals. Those who consumed EDs once a week or more slept for fewer hours per night, achieved lower school attendance, higher Junk Food scores, and exercised more frequently (though the latter effect was only marginally significant) ED consumption alone was not predictive of stress, anxiety or depression at 6-month follow-up. However, in the regression analyses, high stress levels were associated with being a member of the frequent EDs/infrequent breakfast condition

**Table 5:** Qualitative and mixed method studies

Citation	Type	Quality assessment	Country	Participants	Study design	Aims	Main findings
Bunting *et al* (2013)^68^	Full paper	Strong	New Zealand	Boys and girls (n=12) 16–35 years	Focus groups stratified by age (16–21, 22–28 and 29–35 years)	To obtain participants’ perceptions of caffeinated EDs	Themes: advertising, age, alcohol, brand, efficacy, energy-seeking, gender, health, peer influence, product attributes, and safety Taste appeared to be the primary driver motivating the purchase and repurchase of EDs
Costa *et al* (2014)^69^	Full paper	Strong	Australia	Boys and girls (n=40) 12–15 years	Focus groups	To explore perceptions, patterns, and contexts of ED use	Themes: knowledge about ED brands and content, ED use, reasons for ED use, physiological effects and influences on ED use Participants were familiar with a range of EDs and most had used them at least once, but had limited knowledge of ED ingredients and could not easily differentiate them from other drink types
Jones (2011)^70^	Full paper	Strong	Australia	Boys and girls (n=95) 12–14 years 15–17 years	Focus groups (separate by age and gender), supplemented with school and online survey data	To explore perceptions and consumption of alcohol EDs (AEDs)	Many participants commenting that they had consumed AEDs, or seen others consuming them. Findings suggest they may be particularly popular among young females Drinking in the 15–17 age group took place predominantly at parties and friends’ houses, as well as at family gatherings Only a small number of survey respondents raised negative consequences of consuming AEDs
O'Dea (2003)^71^	Full paper	Strong	Australia	Boys and girls (n=78) 11–18 years	Focus groups	To explore the type of nutritional supplements and drinks consumed by adolescents, along with reasons for consumption	In the 2 weeks prior to the focus groups, 42.3% of participants had consumed EDs (compared with 54.6% who consumed sports drinks) Reasons for consumption of EDs: energy, taste, sports performance, soft drink substitute, peer group pressure, attractive packaging

### Effects of energy drink consumption

#### Consumption patterns and associations with health-related behaviours

Cross-sectional survey data suggest that the use of energy drinks is patterned by gender, with several studies indicating that boys were more likely to report consumption than girls, and in greater quantities.^29–46^ Larson *et al*^33^ found a significant association between regular consumption and lower frequency of breakfast for girls only, while Bryant Ludden and Wolfson^36^ found that girls were more likely than boys to report expectations around appetite suppression. Patterns of use according to age were less clear cut, with some studies showing that consumption levels increased with age^32^ ^38^ ^40^ ^46–50^ and others demonstrating that the converse was true.^29^ ^43^ ^48^ ^51^ Mixed racial and ethnic patterns were also identified. Martz *et al* found that black students were less likely to consume energy drinks than their white or Hispanic counterparts, whereas other studies have suggested that consumption levels are highest among black, Hispanic and/or Aboriginal students.^44^ ^48^ ^52^ Higher consumption levels were positively associated with being underweight or obese, being from a single parent family, receiving free school meals, having special educational needs and higher spending money.^44^ ^46^ ^48–50^ Young people with higher academic averages, higher sense of coherence, higher levels of parental monitoring and more educated parents were less likely to consume energy drinks.^14^ ^41^ ^48^ ^53^ ^54^

The main health-related behaviours found to be positively and consistently associated with energy drink consumption were use of alcohol and/or binge drinking, smoking or susceptibility to smoking and other substance use.^14^ ^30^ ^33^ ^34^ ^38^ ^39^ ^41^ ^47^ ^55–57^ Recent longitudinal studies have found that the use of energy drinks at baseline predicted either number of drinking days or frequency of alcohol consumption at follow-up (12 or 16 months).^53^ ^58^ Furthermore, young people who consumed alcohol mixed with energy drinks were more likely to feel and be perceived as more intoxicated, and to have greater odds of driving violations and accidents, after controlling for all other factors.^59^ ^60^ Consumption of energy drinks has been linked to sensation-seeking,^29^ ^38^ ^39^ ^53^ ^57^ self-destructive behaviour,^39^ problems with behavioural regulation and metacognitive skills,^61^ and poor lifestyle behaviours,^49^ including regularly eating junk food or fast food.^46^ ^52^ Al-Hazzaa *et al*^62^ found that energy drink use was associated with increased sedentary behaviour and with higher levels of physical activity. This finding was reinforced by Larson *et al*^33^ and Park *et al*,^52^ who found that energy drink use was correlated with hours spent watching TV or playing video games, and Azagba *et al*^48^ and Nowak and Jasionowski,^43^ who found that consumption was more likely among young people who participated in sports, suggesting a link with diverse leisure activities.

#### Detrimental health and other effects

Using a representative sample of Finns aged 12–18 years, Huhtinen *et al*^31^ found that daily use of energy drinks was strongly associated with four health symptoms: headache, sleeping problems, irritation and tiredness/fatigue. Those who used energy drinks several times a day were 4.5 times as likely to experience headaches and 3.5 times as likely to experience sleeping problems, in comparison with those who did not consume these drinks. However, a more recent study found that the relationship between energy drinks and health symptoms was partly mediated through going to bed late.^63^ A similar survey of 10–12 years in Iceland found that prevalence of physical symptoms such as headaches, stomach aches and sleeping problems generally increased with greater energy drink use for boys and girls, although the frequency of these symptoms was less common among boys on all occasions (p<0.001).^42^ Other cross-sectional studies have demonstrated positive correlations between energy drink consumption and sleeping problems,^46^ ^61^ self-reported medical treatment for an injury,^57^ odds of sustaining a recent or lifetime traumatic brain injury^41^ and hyperactivity/inattention symptoms.^45^

The link between adverse health outcomes and ingestion of energy drinks is supported by routinely collected poison centre data from Australia and the USA.^64–66^ However, these are based on self-report data and the numbers are relatively small. For example, 62 children (mean age 36 months) who had accidentally ingested energy drinks were reported to the New South Wales Poisons Information Centre between 2004 and 2010.^65^ Fourteen had symptoms probably related to energy drink consumption—most commonly hyperactivity—and nine required assessment in hospital. In the USA, 4854 calls (0.2%) received by the National Poison Data System in 2010–2011 were for energy drink exposure cases.^64^ Almost half (46%) were under 6 years, but older children reported the largest proportion of moderate or major effects, such as cardiac rhythm disturbances, hypertension and hyperthermia.

#### Impact on sports performance

Of the three experimental studies identified through the review, two measured the effects of a commercially available energy drink on aspects of sports performance in junior athletes. Pre-exercise ingestion of an energy drink significantly improved handgrip force, running pace at high intensity and number of sprints during a simulated match among tennis players,^67^ and enhanced jump performance, mean leg muscle power output, perception of muscle power and perceived endurance among basketball players.^68^ However, it did not have any influence on mean running pace, peak running speed or ball velocity in the first study, or the precision of basketball shots, number of free throws per second or distances covered by the players in the second study. During the simulated tennis match, sweat rate was slightly higher in the experimental group, producing significantly higher dehydration (p<0.05).^67^

### Attitudes towards energy drink consumption

Three major themes emerged through our analysis of the qualitative or mixed method studies and relevant survey results: reasons for use; influences on use and perceived efficacy and impact.

#### Reasons for use

Taste was consistently reported as the primary driver motivating the purchase and consumption of energy drinks, with energy-seeking emerging as an important but secondary driver.^69–72^ Participants involved in sports, particularly boys, reported using energy drinks as stimulants to enhance their sports performance. Others described using energy drinks as an alternative to soft drinks but only when they had enough money, as they were reported to be more expensive. Typical responses included: “Wakes you up, makes you feel alert and it tastes nice”; “It makes me go hyper” and “I drink it before soccer and I don't lose energy as fast”.^72^ Jones^71^ explored perceptions of alcohol-energy drinks (AEDs) among 12–17 years and suggested that young people liked them because they increased the ‘fun’ at parties and acted as a ‘pick me up’. They also found that the packaging of AEDs (to look like soft drinks) was a factor, particularly for younger teenagers and girls.

#### Influences on use

Advertising and brand loyalty emerged as major influences on young people's use of energy drinks, with participants reporting seeing them advertised on TV, the internet, through games promotions, via sports sponsorship and in shops. In a focus group study involving three age groups (16–21, 22–28 and 29–35 years), industry marketing was seen to target specific drinks at men or women, using sexualised imagery and humour.^69^ Participants in the youngest age group appeared to be more conscious than those in the older groups of the social image they were portraying in their choices, as well as being more sensitive to peer influences when making purchasing decisions. Social situations—and spending time with friends—provided a common context for energy drink consumption across the studies. Parents also played a key role in influencing participants' use of energy drinks, either by disapproving and prohibiting or encouraging and endorsing their use.^70^ It was generally agreed that energy drinks were easily accessible, from convenience stores or supermarkets, provided by parents, shared by siblings or friends, or obtained free at sponsored events.

#### Perceived efficacy and impact

Energy drinks were perceived to confer various beneficial effects on young people's bodies and their sports performance. Participants in one study described short-term health benefits, prevention of illness, improved immunity and a desire to rectify a poor diet.^72^ Others described scenarios where adults used energy drinks to effectively alleviate tiredness related to work, travel or family commitments, leading Costa *et al*^70^ to suggest that these drinks were ‘normalised and perceived as necessary to meet the demands of a busy lifestyle’ (p. 187). Few participants across the studies raised any negative or harmful effects, suggesting young people were either unaware of, or chose to ignore, the possible risks. Negative consequences associated with using AEDs were perceived to relate to the inclusion of a stimulant and depressant in one drink, and difficulties sleeping after consumption.^71^ In the study by Bunting *et al*,^69^ more negative connotations became apparent in the older, adult age groups (22–28 and 29–35 years), who displayed greater scepticism as to whether energy drinks were safe. However, concerns regarding sugar content emerged across all groups and moderation was stressed due to the perceived risks of overconsumption, as opposed to general consumption of energy drinks. The youngest age group (16–21 years) believed these drinks were safe as they would not be on sale if caffeine levels were too high. Findings from other studies highlight limited knowledge of the ingredients of energy drinks, particularly among younger participants.^70^ ^71^

## Discussion

### Summary of principal findings

This review set out to examine evidence of any associations between children and young people's health and well-being, social, behavioural and educational outcomes, and their consumption of energy drinks. It also sought to explore consumer experiences and attitudes towards these drinks.

The evidence demonstrates that the use of energy drinks by children and young people is associated with a number of adverse outcomes and health-damaging behaviours. A total of 410 studies were located, with 46 meeting our inclusion criteria. Two randomised controlled trials demonstrated that pre-exercise ingestion of an energy drink had a positive impact on some aspects of sports performance. However, both studies involved small numbers of elite junior athletes and the results should therefore be treated with some caution. Several cross-sectional studies indicated that energy drink use by children and young people was strongly and positively associated with higher rates of smoking, alcohol and other substance use, as well as being linked to physical health symptoms such as headaches, stomach aches, hyperactivity and insomnia. Two studies provided some evidence of a dose–response effect, although none of the studies was able to determine causality. Use was found to be patterned by gender, with boys consuming more energy drinks than girls, and also by age, although there was some disagreement between studies on the direction of the association. Interestingly, the highest consumption levels have been observed in sedentary individuals and in physically active individuals, suggesting a link with sport as well as screen-based leisure activities. Previous qualitative studies have reported perceived beneficial effects on young people's bodies and sports performance, with little mention of any negative effects and limited knowledge of energy drink ingredients among participants. Taste and energy-seeking were identified as key drivers for consumption. Advertising and brand loyalty have been highlighted as major influences on young people's attitudes towards energy drinks, and peers, parents and siblings were also found to play an important role.

### Comparison with other studies

This is the first comprehensive review of the scientific literature to focus exclusively on evidence relating to the consumption of energy drinks by children and young people. Previous reviews have tended to examine the benefits and risks associated with specific energy drink components, such as sugar or caffeine. These studies provide important insights but fail to account for the fact that the presence of other substances such as guarana, ginseng and taurine in variable quantities may generate uncertain interactions and exacerbate any risks.^73^ In addition, there is an established biochemical interaction between energy drink contents and alcohol, resulting in physical and psychological side effects and increased risk-taking behaviour.^74^ ^75^ The aforementioned EFSA study found that more than half of young energy drink consumers (53%) reported co-consumption with alcohol.^3^ Qualitative studies suggest that young adults use energy drinks to continue partying and drinking alcohol over a longer period, and that they may experience negative effects ranging from difficulty sleeping to being admitted to hospital.^76^ ^77^ Further evidence is provided by the numerous clinical case reports relating to young people receiving emergency treatment for overconsumption of energy drinks with or without alcohol in recent years (for examples, see:^78–82^).

Consumption of sugar-sweetened beverages by children and young people has been shown to result in greater weight gain, increased body mass index and higher incidence of type 2 diabetes.^18^ ^83^ A review on the suitability of caffeinated drinks for children found that high caffeine intakes (>5 mg/kg body weight per day) were associated with an increased risk of anxiety and withdrawal symptoms.^84^ However, evidence from adult studies and expert panels was used to suggest that relatively small amounts of caffeine may benefit cognitive function and sports performance, as well as contributing to daily fluid intakes. Furthermore, the author was a paid member of the Tea Advisory Panel and the work was funded by the UK Tea Council. Several other studies located through our review were funded by the soft drinks industry or conducted by researchers with acknowledged conflicts of interest. Previous independent reviews on energy drinks highlight a number of implications for children's health and well-being, although they also draw on expert opinion and adult studies.^12^ ^13^ ^24–27^ A report by the Committee on Nutrition and the Council on Sports Medicine and Fitness in the US raises concerns about the unintentional (through the use of energy drinks for rehydration) and intentional (through the use of energy drinks to combat fatigue) ingestion of potentially large amounts of caffeine and other stimulant substances.^12^ They suggest that paediatricians have a role to play in educating children and parents on the differences between sports and energy drinks, and to counsel that routine ingestion of sugar-sweetened beverages should be avoided or restricted.

### Strengths and limitations of the study

The strengths of our review include the comprehensiveness of our searches, the systematic study selection process and rigorous synthesis methods used. The review was undertaken by a multidisciplinary team of independent academic researchers. The inclusion of qualitative research exploring children and young people's views on energy drinks, alongside quantitative studies on health and other effects associated with their consumption, helps to enhance the relevance of the findings for the design and evaluation of future policy and practice interventions.

As with any literature review, the strength of our conclusions is limited by the quality of the individual studies, which varied. The small sample sizes in the experimental and retrospective studies, reliance on self-report data in many of the observational studies and small number of qualitative studies located are all limitations of the review. Very few of the included studies examined educational or social outcomes, highlighting a need for further research that examines the short-term and long-term impact of energy drinks in relation to a wider range of outcomes. Almost half of the studies were conducted in North America and most involved high school students rather than younger children. Owing to time and resource constraints, we excluded non-English language publications and may not have identified all unpublished studies.

### Conclusion and policy implications

This review adds to the growing evidence base on the health effects associated with energy drink consumption and suggests that there may be more negative than positive implications for children and young people. However, factors such as taste, brand loyalty and perceived beneficial effects help to enhance their popularity among young consumers. Consideration of the patterns and reasons for energy drink consumption identified in this review may help future interventions to ensure appropriate behaviours are targeted and are relevant to the population. Gender was identified as an important factor, in combination with gendered marketing and perceived links to sports performance, particularly among boys. The challenge for policies and interventions that seek to address this issue is to recognise the complexities of children and young people's consumption choices. Although health education targeting individuals is unlikely to achieve a substantial impact, definitive information about the safety of energy drink consumption should be provided by healthcare and other professionals, who may in turn need guidance, for example, from the UK National Institute of Health and Care Excellence (NICE). More research is needed to explore the longer-term health impacts, given that childhood and adolescence are critical yet understudied periods in the development of health-related behaviours. The potential effects of heavy and long-term energy drink consumption on child development, behaviour and educational outcomes also warrant further study.
